# Evaluation of post-laryngectomy dysphagia rehabilitation using High-Resolution impedance manometry: an exploratory study

**DOI:** 10.1007/s00405-025-09642-z

**Published:** 2025-10-03

**Authors:** Marise Neijman, Maarten J. A. van Alphen, Rob J.J.H. van Son, Martijn M. Stuiver, Frans J.M. Hilgers, Michiel W.M. van den Brekel, Lisette van der Molen

**Affiliations:** 1 Department of Head and Neck Oncology and Surgery, the Netherlands Cancer Institute, Plesmanlaan 121, Amsterdam, 1066 CX the Netherlands; 2 Amsterdam Center for Language and Communication (ACLC), University of Amsterdam (UvA), Binnengasthuisstraat 9, Amsterdam, 1012 ZA the Netherlands; 3 Center for Quality of Life and Division of Psychosocial Research and Epidemiology, Netherlands Cancer Institute, Plesmanlaan 121, 1066 CX Amsterdam, the Netherlands; 4 Division of Epidemiology and Data Science, Amsterdam UMC, Cancer Center Amsterdam, De Boelelaan 1118, 1081 HV Amsterdam, Netherlands; 5 Department of Oral and Maxillofacial Surgery, Amsterdam UMC, Meibergdreef 9, Amsterdam, 1105 AZ the Netherlands; 6 Netherlands Cancer Institute - Antoni van Leeuwenhoek, Plesmanlaan 121, 1066 CX Amsterdam, the Netherlands

**Keywords:** Total laryngectomy, Swallowing rehabilitation, Pharyngeal pressure, High Resolution (Impedance) manometry, Swallowing exercise aid

## Abstract

**Aim:**

This exploratory study analyzes High-Resolution Impedance Manometry (HRIM) data obtained during a phase II rehabilitation trial in laryngectomees at baseline (T0), after six-weeks of resistance-based training (T1), and after eight weeks of rest (T2), exploring its potential value in alaryngeal dysphagia research and clinical practice.

**Methods:**

Pharyngeal HRIM was combined with videofluoroscopy to evaluate swallowing biomechanics in 17 laryngectomy patients. Parameters included Pharyngeal (Velo-, Meso-, and Hypopharyngeal) Contractile Integrals, Intra-Bolus Pressure, UES Relaxation Time, Maximum Admittance, and Integrated Relaxation Pressure.

**Results:**

No significant differences were found in the Pharyngeal Contractile Integrals, Intra-Bolus Pressure, Relaxation Time, Maximum Admittance, or Integrated Relaxation Pressure. However, pharyngeal pressures and Maximum Admittance slightly decreased from T0 to T1, and increased at T2 for all consistencies (thin, extremely thick, solid).

**Conclusion:**

Alaryngeal resistance-based swallowing exercises lead to small, non-significant differences in pharyngeal pressures. HRIM provides valuable insights, but its application for this population remains uncertain.

**Supplementary Information:**

The online version contains supplementary material available at 10.1007/s00405-025-09642-z.

## Introduction

Swallowing problems following total laryngectomy are common. Dysphagia affects up to 72% of patients, and often results in significant physical and psychosocial challenges [1–4]. Laryngectomized individuals with dysphagia frequently attempt to compensate for their difficulties by omitting or modifying certain food consistencies, or by using techniques such as liquid wash or prolonged chewing [5]. Also, they often experience anxiety and avoid social interactions, which can negatively impact their quality of life [3]. Despite the well-known consequences of post-laryngectomy dysphagia, this topic only has received limited attention in clinical research [6]. This is likely due to the frequency of other important handicaps, the scarcity of effective rehabilitation options, and the fact that aspiration, the harshest element of “regular” dysphagia, does not play a role in laryngectomy patients. This latter aspect is underlined by the finding that in a cohort of 20 laryngectomized individuals with self-reported dysphagia, their BMI was normal and they have no aspiration pneumonias [5].

Recently, we reported on an effective post-laryngectomy dysphagia rehabilitation program, which is based on sport-medical principles of muscle strengthening [5]. These principles imply that for effective muscle strengthening, one must regularly practice against a resistance of 60–70% of the 1-RM (one-repetition maximum: the maximum force generated in a single maximal contraction), and that with increasing strength this resistance should be adjusted accordingly [7, 8]. In that clinical phase II rehabilitation trial with the aforementioned 20 laryngectomized individuals, we could demonstrate that a 6-weeks training program with an adjustable resistance tool (the Swallowing Exercise Aid 2.0 (SEA2.0) leads to a significant objective and subjective reduced dysphagia and improved quality of life [5]. The positive results from that study were still noticeable six months later, even though most participants did not continue training [9].

To further understand the biomechanics and physiological effects of the above-mentioned rehabilitation program, we applied pharyngeal High-Resolution Impedance Manometry (HRIM). This pharyngeal HRIM assessment is a relatively new technique that can help objectify swallowing biomechanics, including pressure dynamics and bolus flow [10–12]. HRIM has gained increasing interest and advancements over the past few decades [13]. A scoping review, specifically focusing on the use and potential value of HRIM in head and neck cancer patients, including three studies with total laryngectomy patients, showed limited use thus far, but also some clinical potential, for instance to evaluate the effects of swallowing therapy over time [14]. The present paper analyzes the HRIM data obtained during the phase II clinical rehabilitation trial at baseline (T0), after 6-weeks training of resistance-based swallowing exercises (T1), and after eight weeks of rest (T2), and correlate the measurements with objective and subjective outcomes related to swallowing and thus to explore its potential value in alaryngeal dysphagia research and clinical practice after total laryngectomy.

### Methods

This study is part of a clinical phase II trial which was approved by the Medical Ethical Committee (METC21.0904/N21STL) [5]. The guidelines of the Helsinki Declaration were followed, and written informed consent was obtained from each participant before inclusion. Data was collected at the Head and Neck Oncology & Surgery Department of a tertiary National Cancer Institute.

### Participants

Between April 2022 to February 2023, laryngectomized individuals with self-reported dysphagia were recruited from the own institute and via the Dutch Patient Association for Head and Neck (PVHH). Participants had to be at least six months post-surgery and radiotherapy before enrollment. Pharynx reconstruction method and history of stenosis and dilatation was not a selection criterion. Of the 20 participants in the clinical phase II trial, HRIM assessment was possible in 19 individuals (16 male) with a median age of 70 years (range 45–77). One participant (S19) had to be excluded due to anxiety for the HRIM catheter. Table 1 shows the characteristics of the 19 participants in the HRIM study.

**Table 1:** Participant characteristics

Participant			Tumor		Treatment					
	Sex	Age	Location	TNM	Indication	Months since TL	Pharynx closure	Myotomy	Stenosis (dilatations)	Timing (C)RT
S01	M	78	Hypopharynx	T1N1	Functional	47	T/Y	No	No (0)	Pre-surgery
S02	M	65	Hypopharynx	pT4aN0	Curative	73	PM	Yes	Yes (2)	Post-surgery
S03	M	75	Larynx	cT2N0	Salvage	75	T/Y	Yes	Yes (1)	Pre-surgery
S04	M	73	Hypopharynx	cT3N2b	Curative	40	PM	No	Yes (1)	Pre-surgery
S05	F	62	Trachea	pT4bN0	Curative	35	Horizontal	Yes	No (0)	Post-surgery
S06	M	53	Larynx	pT4N2a	Curative	12	T/Y	Yes	No (0)	No
S07	M	72	Larynx	T1bN0	Salvage	94	Vertical	Yes	No (0)	Pre-surgery
S08	M	71	Larynx	cT2N0	Salvage	44	PM	Yes	No (0)	Pre-surgery
S09	M	76	Hypopharynx	T3N2c	Curative	24	ALT	No	Yes (2)	Post-surgery
S10	M	61	Larynx	cT4aN0	Curative	47	T/Y	Yes	No (0)	No
S11	M	67	Larynx	cT4aN0	Curative	65	T/Y	Yes	No (0)	No
S12	M	77	Larynx	T4N0	Salvage	274	T/Y	Yes	No (0)	Pre-surgery
S14	F	45	Larynx	cT3N0	Salvage	22	T/Y	Yes	Yes (2)	Pre-surgery
S15	M	50	Hypopharynx	pT4N3b	Curative	33	SCAIF	No	No (0)	Post-surgery
S16	M	66	Larynx	pT4aN0	Curative	16	T/Y	Yes	No (0)	Post-surgery
S17	M	63	Larynx	T2N0	Salvage	67	Vertical	Yes	No (0)	No
S18	M	70	Larynx	rT2N0	Salvage	9	T/Y	No	No (0)	Post-surgery
S20	M	77	Larynx	T4aN0	Salvage	138	Vertical	Yes	No (0)	Post-surgery
S21	F	73	Hypopharynx	T4aN0	Salvage	87	Gastric Pull-up	No	No (0)	No
**Median (Range)**		**70 (45-78)**				** 47 (9-274)**				

Abbreviations: TNM, a classifying system for malignancy consisting of T (tumor) N (node) and M (metastasis); PM, Pectoralis Major Flap; ALT, Anterolateral Thigh Flap; SCAIF, Supraclavicular Artery Island Flap; (C)RT, (Chemo) Radiation Therapy. This table, adapted from the previously published study [5], has been modified to exclude participant S19. S19 was removed due to anxiety to the HRIM catheter

### Exercise protocol

The SEA2.0 device provides quantifiable adjustable resistance training for swallowing and jaw exercises, and audible and tactile feedback. Exercises included the Chin Tuck (CTAR), Jaw Opening (JOAR) and Effortful Swallow (ESAR) Against Resistance [5]. To obtain the training load, the initial resistance was set at 60–75%1RM as measured with a digital dynamometer (MicroFET™, Biometrics, Almere, the Netherlands) device and was then adjusted to achieve a load that was rated as at least strenuous at 30 repetitions in a first practice round. For the CTAR and JOAR exercises, the 6-weeks exercise protocol consisted of an isokinetic and isometric part. During the isokinetic part, the participant was asked to perform the exercise 30 times. During the isometric part, the participant performed the exercise three times for 60 s, with at least 60 s of rest between the sets. The ESAR exercise was performed ten times consecutively after another 60 s of rest. The total duration of the exercises was estimated to be 15–20 min per session. All participants were instructed to perform the SEA 2.0 exercises three times a day, seven days a week, for six weeks in total. They received a written instruction sheet with pictures before starting their ‘six-week training period’ and were followed up every one or two weeks. Participants were instructed to decrease the resistance or stop the exercises if they felt discomfort or pain in the chest, chin, neck or in/around their temporomandibular joint during or after the exercises. Patients were evaluated before the training (T0), after the six-week training (T1), and after eight weeks of rest thereafter (T2).

The outcomes of the applied objective and subjective measurements relevant for the present analyses are summarized here. Chin Tuck and Jaw Opening strength assessments (in newton (N), using a dynamometer mounted in a headframe) showed that muscle strength (significantly) increased around 50% [5, 15]. Swallowing capacity (using the SPEAD test) significantly increased from 2.4 to 3.8 g per second [5, 16]. Swallowing efficiency (using Videofluoroscopic Swallowing Studies (VFSS) and the %Residue Scale of the Dynamic Imaging Grade of Swallowing Toxicity (DIGEST) scoring tool) improved from 2 (‘*majority residue’*) to 1 (‘*Less than half residue’*) [5, 17].

Subjective improvements were seen on the MD Anderson Dysphagia Inventory (MDADI) questionnaire (69.8 to 76.9), the EAT10 questionnaire (10.6 to 8.0), and the Swallowing Outcomes After Laryngectomy (SOAL) questionnaire (13.6 to 11.0) [5, 18–22].

### High-Resolution impedance manometry

To investigate the biomechanics of swallowing, real-time pharyngeal pressures in mmHg during swallowing were assessed using HRIM [10]. The Solar GI HRIM Solid State Trolley System (SN 18710476, Laborie, Enschede, the Netherlands) was combined with a 36-sensor solid-state catheter (Unisensor HRM K103659-E-118-D, Laborie, Enschede, the Netherlands). The assessment took place on the radiology department and was combined with VFSS.

**Fig. 1:**
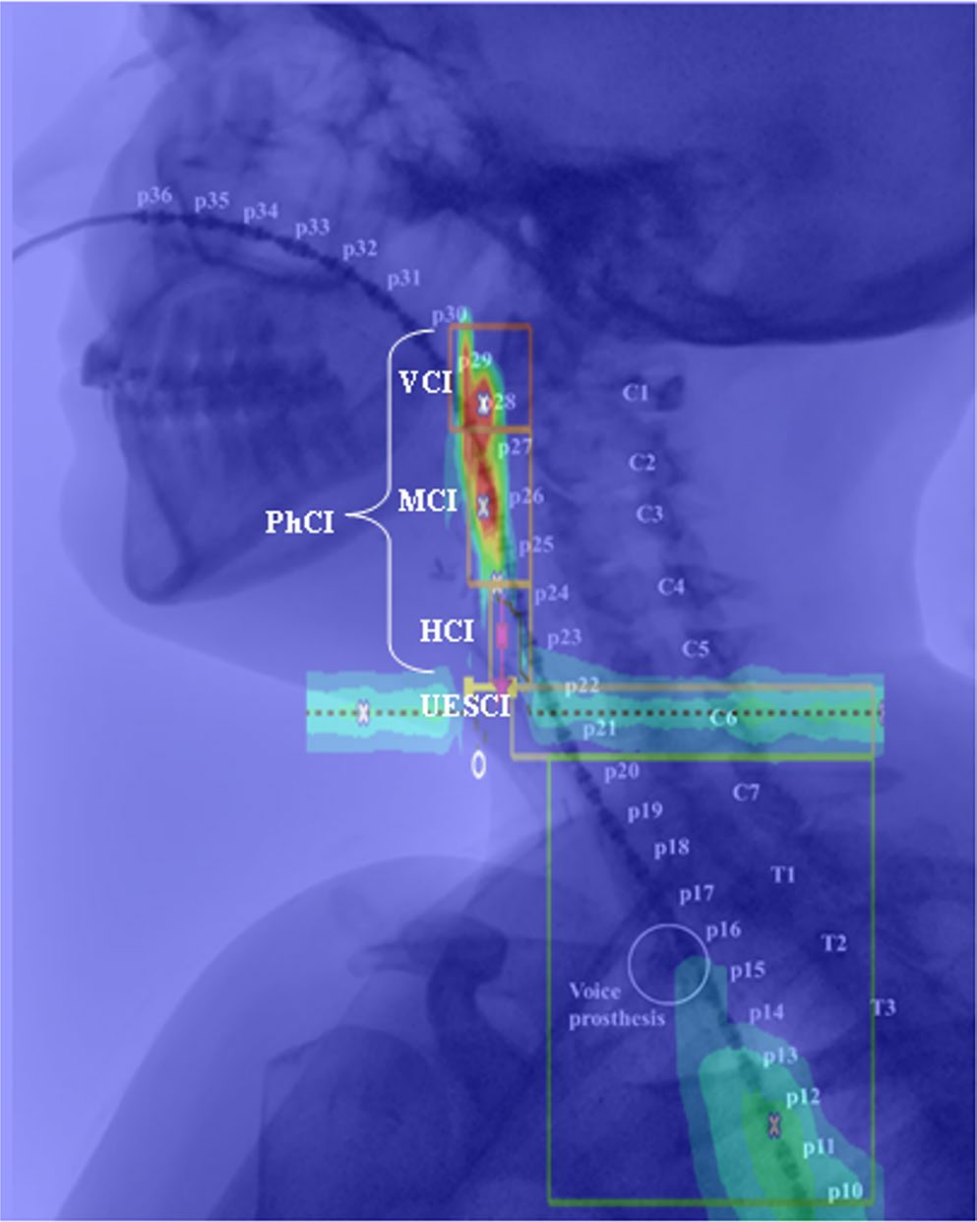
Videofluoroscopic image showing a participant with the High-Resolution Impedance Manometry catheter in place. The sensors are numbered, and the color plot is overlaid, illustrating the different regions of interest (ROIs) from top to bottom: Velopharyngeal Contractile Integral (VCI), Mesopharyngeal Contractile Integral (MCI), Hypopharyngeal Contractile Integral (HCI), Upper Esophageal Sphincter Contractile Integral (UES CI)

Prior to the HRIM assessment, the participants, sitting in upright position, were asked to select their preferred nostril for catheter insertion, which then was anesthetized with lidocaine spray. After approximately five minutes, the catheter was inserted via the nose into the pharynx to a depth of 36 cm and secured on the nose with nasofix tape. The participant rested for approximately five to ten minutes to get used to the catheter in their nose and pharynx and the have the lidocaine work out. Meanwhile, the radiology technician calibrated the CombiDiagnost R90 (Philips, Best, the Netherlands) and the participant was instructed to move the entire body (keeping the body in an upright sitting position) approximately 20 degrees rotated to the right, creating a slightly angled view to improve residue detection, see Fig. 1.

The participant was then instructed to swallow multiple consistencies of different bolus sizes of a combination of Omnipaque™ 300 mg/ml and the Standardized Bolus Medium (SBMkit) product (Trisco, Pty. Ltd., Australia) in a random order. The order was randomized to minimize the effects of a potential learning curve. Consistencies included were thin, extremely thick, and solid boluses, all of which met the requirements of the International Dysphagia Diet Standardization Initiative (IDDSI) [23]. In total, the participant swallowed two times 10 cc boluses of thin (IDDSI 0) and extremely thick (IDDSI 4) liquids, via a syringe. Additionally, they had to swallow a contrast-coated cracker (IDDSI 7).

The raw pressure data was exported to an ASCII (.asc) format containing no identifiable information and uploaded for analysis to the web-based platform SwallowGateway™ (www.swallowgateway.com, version 2022; Flinders University, Adelaide, Australia), an analysis tool that has been proven reliable in non-laryngectomees [24]. The software automatically converted the raw impedance (Ohms, Ω) values to admittance (millisiemens, mS) for bolus presence/distension.

Individual swallows were selected by drawing a region of interest (ROI) from the velopharynx to the esophageal transition zone. In case of boluses involving multiple swallows, the swallow with the highest impedance was chosen for analysis. For each selected individual swallow, the Upper Esophageal Sphincter (UES) was first identified on the color plot. Markers were then systematically placed: the hypopharynx (lower neo-pharynx) marker about 2 cm above the UES, the mesopharynx (higher neopharynx) 3–4 cm above the hypopharynx, and the velopharynx roughly 2 cm above the mesopharynx. These markers were compared to the VFSS and adjusted if necessary. This method defined the pharynx from the base of the skull to the C6 vertebra, as shown in Fig. 1.

Various HRIM core metrics were automatically generated via the SwallowGateway™ software, including the *Pharyngeal Lumen Occlusive Pressure*, *Hypopharyngeal Intra-Bolus Distension Pressure*, and *UES Relaxation and Opening *(see Fig. 1; Table 2) [11]. For clarity, detailed descriptions and definitions of these HRIM core metrics are provided in Appendix 1.

**Table 2:** Definitions and interpretation of pharyngeal High-Resolution impedance manometry [11, 25, 26, 27, 28, 29, 30, 31, 32, 34, 43]

Metric class	Metric	Acronym	Unit	Interpretation
Pharyngeal Lumen Occlusive Pressure	Pharyngeal Contractile Integral	PhCI	mmHg.cm.s	Decreased values indicate weakness of the whole pharyngeal and/or subcomponents of the velo-, meso- and/or hypopharyngeal regions
	Velopharyngeal Contractile Integral	VCI	mmHg.cm.s	
	Mesopharyngeal Contractile Integral	MCI	mmHg.cm.s	
	Hypopharyngeal Contractile Integral	HCI	mmHg.cm.s	
Hypopharyngeal Intra-Bolus Distension Pressure	Hypopharyngeal Intra-Bolus Pressure	IBP	mmHg	Increased value indicate resistance at the UES
UES Relaxation & Opening	UES Integrated Relaxation Pressure	UES IRP	mmHg	Increased value indicate UES restriction and impaired relaxation
	UES Relaxation Time	UES RT	s	Decreased value indicate reduced UES opening duration
	UES Maximum Admittance	UES MaxAd	mS	Decreased values indicate reduced UES opening extent

Abbreviations: mmHg, millimeters of Mercury; cm, centimeter; s, seconds; mS, miliseconds; UES, Upper Esophageal Sphincter

### Correlations

Correlations were calculated to understand whether there is a relationship between the HRIM outcomes and the previously published objective outcomes of the strength measurements (Chin Tuck and Jaw Opening), swallowing capacity (SPEAD test), swallowing efficiency (DIGEST) and subjective outcomes (MDADI, SOAL, and EAT10 questionnaires) [5].

### Statistical analysis

The software program R (version 4.2.1) was used for all statistical analyses. Descriptive statistics were used for participant characteristics. To summarize the changes over time of the continuous outcomes, robust Linear Mixed-Effects Models (robust LME [35]) were used, with time considered as a categorical variable. Robust LME models were chosen because an initial analysis showed the presence of outliers in the data. The robust LME method is usable in small samples containing outliers or other contaminations, and takes the three different time points (T0, T1 and T2) in account. The estimated marginal means from the model, with a corresponding 95% confidence interval (95%CI) were plotted along with the individual data.

Correlations were calculated using Spearman and displayed in a heat map for comprehensive visualization for T0, T1 and T2. Correlations were interpreted as negligible (0.00-0.19), low (0.20–0.39), moderate (0.40–0.59), high (0.60–0.79), and very high (0.80-1.00). Because of the small sample size of the study, we did not consider statistical significance levels.

## Results

**Fig. 2:**
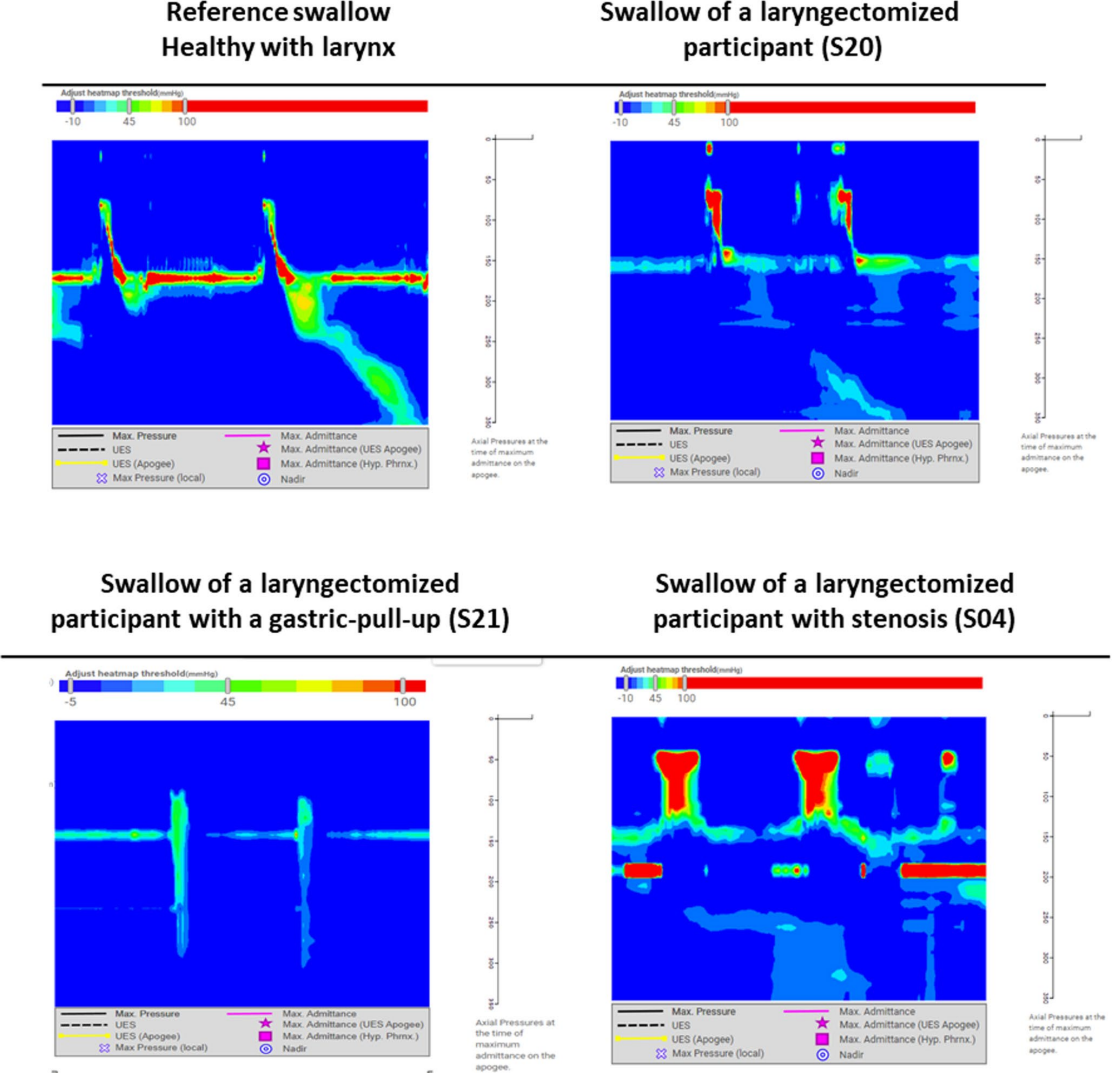
Color plots of High-Resolution Impedance Manometry during 10 cc of thick liquid (IDDSI 4) swallows of reference (healthy) and three different participants, analyzed with SwallowGateway^™^

For the analysis of the HRIM data two participants had to be excluded. Participant S21, which was the only participant with a Gastric Pull-Up, was excluded because her “pharyngeal” anatomy and the HRIM color plots were incomparable to the others (see Fig. 2). Participant S01 had to be excluded from the analysis due to missing data at T0 (unable to swallow), T1 (due to technical issues) and T2 (incomplete data).

Although there were some missing data for participants S14 and S12, they could remain in the analysis. Participant S14 had missing data at T0 (due to a technical issue), but it was still possible to compare the T1 and T2 data. And for participant S12, the T2 data could not be collected due to unrelated health issues, but it was still possible to compare the T0 and T1 data.

**Fig. 3:**
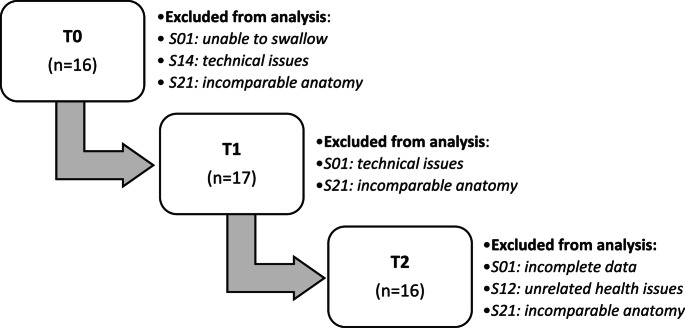
Flowchart of participants included in the analysis

Participant S04 suffered from stenosis, which is clearly visible in the variations in pressure patterns in the color plots in Fig. 2. Since he preferred training with the SEA over undergoing dilation, he remained included in the study. Thus, in total data analysis was done for 17 patients, see the flowchart in Fig. 3.

### High-Resolution impedance manometry

All available LME outcomes of the HRIM metrics at T0, T1, and T2 are presented in Table 3. A distinction is made between the different consistencies: thin liquid (IDDSI 0), extremely thick liquid (IDDSI 4), and solid (IDDSI 7).

**Table 3:** High-Resolution impedance manometry core metrics of swallowing

HRIM core metrics		T0 (*n* = 16)	T1 (*n* = 17)	T2 (*n* = 16)
		PMM (95%CI)	PMM (95%CI)	PMM (95%CI)
10 cc thin				
PhCI	in mmHg.cm.s	242 (175, 309)	210 (144, 277)	235 (168, 302)
VCI	in mmHg.cm.s	96 (62, 130)	78 (45, 111)	90 (57, 124)
MCI	in mmHg.cm.s	102 (66, 139)	91 (55, 127)	106 (69, 142)
HCI	in mmHg.cm.s	35 (18, 52)	34 (17, 50)	36 (19, 53)
UESIRP	in mmHg	2.5 (−0.5, 5.5)	2.0 (−0.9, 4.9)	2.8 (−0.2, 5.8)
UESRT	in s	0.4 (0.3, 0.5)	0.5 (0.3, 0.6)	0.5 (0.3, 0.6)
UESMaxAd.	in mS	1.8 (1.4, 2.1)	2.0 (1.6, 2.3)	1.6 (1.3, 2.0)
BPT	in seconds	1.1 (0.7, 1.5)	0.8 (0.4, 1.2)	1.2 (0.8, 1.6)
IBP	in mmHg	155 (88, 222)	165 (99, 231)	154 (87, 221)
10 cc thick				
PhCI	in mmHg.cm.s	286 (217, 355)	230 (162, 299)	266 (197, 336)
VCI	in mmHg.cm.s	108 (67, 149)	78 (38, 118)	102 (61, 142)
MCI	in mmHg.cm.s	137 (102, 172)	104 (70, 139)	114 (80, 149)
HCI	in mmHg.cm.s	37 (19, 54)	37 (20, 54)	46 (28, 63)
UESIRP	in mmHg	5.5 (2.1, 9.0)	3.9 (0.6, 7.3)	4.4 (1.0, 7.9)
UESRT	in s	0.4 (0.3, 0.5)	0.4 (0.3, 0.5)	0.4 (0.3, 0.5)
UESMaxAd.	in mS	2.1 (1.7, 2.5)	2.1 (1.7, 2.5)	2.0 (1.6, 2.4)
BPT	in seconds	0.9 (0.5, 1.4)	0.9 (0.4, 1.3)	1.2 (0.8, 1.7)
IBP	in mmHg	156 (93, 219)	145 (82, 207)	159 (97, 222)
Solid				
PhCI	in mmHg.cm.s	268 (190, 347)	207 (130, 284)	229 (150, 307)
VCI	in mmHg.cm.s	90 (58, 123)	66 (34, 97)	71 (39, 104)
MCI	in mmHg.cm.s	114 (73, 155)	94 (53, 134)	104 (63, 145)
HCI	in mmHg.cm.s	46 (25, 66)	39 (19, 59)	50 (30, 71)
UESIRP	in mmHg	6.2 (1.4, 11.1)	5.5 (0.8, 10.2)	9.1 (4.2, 13.9)
UESRT	in s	0.4 (0.3, 0.5)	0.3 (0.2, 0.4)	0.4 (0.3, 0.5)
UESMaxAd.	in mS	2.0 (1.7, 2.4)	2.3 (2.0, 2.7)	2.4 (2.1, 2.8)
BPT	in seconds	1.4 (0.2, 2.6)	1.9 (0.7, 3.0)	1.3 (0.1, 2.4)
IBP	in mmHg	185 (97, 273)	135 (49, 221)	167 (79, 255)

Abbreviations: PMM, Predicted Marginal Mean; HRIM, High-Resolution Impedance Manometry; T0, baseline; T1, short-term results after six weeks of training with the SEA2.0; T2, long-term results after eight weeks of rest; PhCI, Pharyngeal Contractile Integral; VCI, Velopharyngeal Contractile Integral; MCI, Mesopharyngeal Contractile Integral; HCI, Hypopharyngeal Contractile Integral; UES IRP, UES Integrated Relaxation Pressure; UES RT, UES Relaxation Time; UES MaxAd., UES Maximum Admittance; BPT, Bolus Presence Time; IBP, Intra Bolus Pressure; mmHg, Millimeters of Mercury; cm, centimeters; s, seconds.

#### Pharyngeal lumen occlusive pressure

**Fig. 4:**
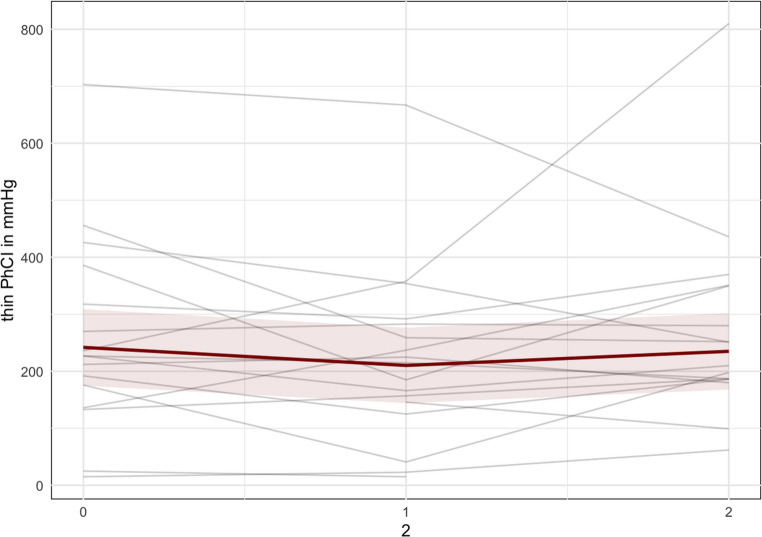
Pharyngeal Contractile Integral (PhCI) measured during swallowing of thin liquid 10 cc bolus. Each gray line represents one participant, while the red line represents the predicted marginal mean from the LME model, with the pink shading indicating the 95% confidence interval. The illustration of the trend that has been found. In Appendix 2, individual patient graphs can be found

No significant changes were observed in the pharyngeal lumen occlusive pressure measurement; however, a trend was noted where the pharyngeal contractile integral (PhCI) pressure decreased from T0 to T1 and then increased at T2 for both thin liquid, thick liquid and solid boluses (see Fig. 4). For instance, during 10 cc thin liquid boluses, the PhCI decreased from 242 mmHg (95%CI 175, 309) at T0 to 210 mmHg (95%CI 144, 277) at T1, and then increasing to 235 mmHg (95%CI 168, 302) at T2 (see Table 3).

#### Hypopharyngeal intra-bolus distention pressure

No significant changes were observed in the hypopharyngeal intra-bolus pressure (IBP) measurement; however, a trend was noted where the pressure increased from T0 to T1 and then decreased at T2 for thick liquid and solid boluses. For thin liquid boluses, it was the opposite. For instance, during 10 cc thin liquid boluses, the IBP increased from 155 mmHg (95%CI 88, 222) at T0 to 165 mmHg (95%CI 99, 231) at T1, and subsequently decreased to 154 mmHg (95%CI 87, 221) at T2 (see Table 3).

#### UES relaxation and opening

No significant changes were observed in the UES Relaxation and Opening measurement; however, a trend was noted where the UES IRP, a measure for the extent of UES relaxation, decreased from T0 to T1 and then increased at T2 for both thin liquid, thick liquid and solid boluses. For instance, during 10 cc thin liquid boluses, the average UES IRP decreased from 2.5 mmHg (95%CI −0.5, 5.5) at T0 to 2.0 mmHg (95%CI −0.9, 4.9) at T1, and then increased to 2.8 mmHg (95%CI −0.2, 5.8) at T2. The UES RT and UES MaxAd remained stable over time (see Table 3).

### Correlations

**Fig. 5:**
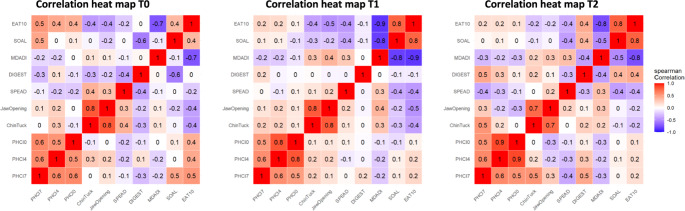
Correlation heat maps of T0, T1 and T2

Figure 5 presents the heat maps of correlations between HRIM metrics at T0, T1 and T2. Notable moderate correlations (above 0.4) of objective outcomes include Jaw Opening and Chin Tuck (0.7 to 0.8). For the HRIM PhCI metric, high correlations were found between PhCI thin, thick and solid (0.5 to 0.9), and moderate between PhCI solid and DIGEST (0.5). No notable correlations were found between the HRIM metrics and the swallowing capacity (SPEAD), or muscle strengths (Chin Tuck and Jaw Opening).

Moderate correlations (above 0.4) of subjective improvements of swallowing were found between the EAT10 and SOAL (0.8), EAT10 and MDADI (−0.7 to −0.9), EAT10 and Jaw Opening (−0.5), EAT10 and PhCI (0.5), SOAL and MDADI (−0.5 to −0.8), SOAL and DIGEST (−0.6), and between SOAL and PhCI solid (0.5).

At participant level, ten participants (S02, S05, S07, S08, S09, S10, S12, S16, S17, S20) showed lower PhCI (meaning global weakness) and improvements in swallowing capacity (SPEAD test) and muscle strength (Chin Tuck or Jaw Opening) at T1 compared to T0, see Table 4.

**Table 4:** Overview of increase or decrease of PhCI thin, SPEAD, and muscle strength (Chin tuck and jaw Opening) at participant level

																		Count		
T1 vs. T0	S02*	S03*	S04*	S05	S06	S07	S08	S09*	S10	S11	S12	S14*	S15	S16	S17	S18	S20	↑	↓	NA
PhCI thin	↓	↑	↓	↓	↑	↓	↑	↓	↓	↑	↓	NA	↑	↓	↓	↑	↓	6	10	1
SPEAD	↑	↑	↓	↑	↑	↑	↑	↑	↑	↑	↑	↑	↑	↑	↑	↑	↑	16	1	0
ChinTuck	↑	↑	↑	↑	↑	↑	↑	↑	↑	↑	↑	↑	↓	↑	↑	↑	↑	16	1	0
JawOpening	↑	↓	↑	↑	↓	↑	↑	↑	↑	↓	↓	↓	↓	↑	↑	↑	↑	11	6	0

The PhCI was used as it represents extremely thick and solid consistencies. Ten participants (S02, S05, S07, S08, S09, S10, S12, S16, S17, S20) showed lower PhCI (meaning global weakness) and improvements in swallowing capacity (SPEAD test) and muscle strength (Chin Tuck or Jaw Opening) at T1 vs. T0

Abbreviations: NA, HRIM measurement not available; *Participants with history of stenosis and dilatations.

## Discussion

Measuring pharyngeal pressure and swallowing dynamics using HRIM after laryngectomy is challenging. We found that occlusive intraluminal pressure parameters are minimally, and not significantly changed by training with the SEA2.0 in spite of the fact that muscle strengths of other muscles in the neck are increased and swallowing efficiency is improved. This indicates that swallowing efficiency is very multifactorial and pharyngeal muscle strength and pressure are not the only parameters important in swallowing,

### Pharyngeal lumen occlusive pressure metrics

At the different time points, no significant changes were observed in the pharyngeal lumen occlusive pressure measurements. However, in the pharyngeal lumen occlusive pressure metrics (PhCI, VCI, MCI, HCI), a trend of decreasing pressure between T0 and T1 was observed, which returned to or even exceeded baseline (T0) levels at T2. According to literature (see Table 2), such a (slight) decrease should be interpreted as a (slight) increase in weakness in these regions after the resistance-based therapy. This interpretation is not supported by our earlier clinical findings, though, where the same participants showed improvements on the objective (e.g., muscle strength, swallowing capacity and swallowing efficiency) and subjective (PROMs) swallowing function parameters [5]. We therefore hypothesize that, also considering the lack of correlation between manometric outcomes and the objective swallowing data in our patient population (further discussed below), the increased muscle strength (Chin Tuck and Jaw Opening) may allow participants to use lower occlusive intraluminal pressure to swallow the bolus. In other words, their swallowing might be more effective and efficient without requiring higher occlusive intraluminal pressures. However, as the differences in pressure are minimal, it could very well be that our training techniques do not really affect pharyngeal musculature, but other muscles related to swallowing.

To compare our (alaryngopharyngeal) HRIM data with literature about healthy (laryngopharyngeal) HRIM data, we used a paper on 98 healthy individuals (43 males, 56 females; mean age of 51 (SD 21) years) that also included 10 cc thin liquid swallows [36]. Small differences between our laryngectomized participants and the healthy reference values were found. For instance, on thin liquid boluses, laryngectomized participants showed lower VCI (96 vs. 130 mmHg.cm.s) and HCI (35 vs. 48 mmHg.cm.s); higher MCI (102 vs. 76 mmHg.cm.s) and comparable PhCI (242 vs. 240 mmHg.cm.s) metrics. This suggests that the laryngectomized participants in our cohort have reduced pressures in their Velopharynx and Hypopharynx (lower neopharynx), while the Mesopharynx (higher neopharynx) demonstrated elevated pressures, leading to a comparable overall PhCI pressure. The finding that the Velopharynx (VCI) is slightly lower in our study compared to healthy reference values aligns with earlier research, which has shown that laryngectomized patients often suffer from velopharyngeal insufficiency [37]. Also, the lower Hypopharynx (HCI) outcomes in our study align with earlier findings of reduced peak mid-pharyngeal pressures and lower hypopharyngeal (neopharyngeal) peak (contractile) pressures, maybe related to myotomy performed at the time of laryngectomy [38].

When comparing our findings to other studies involving laryngectomized patients, we identified three studies that investigated swallowing pressures with 10 cc thin liquid boluses in this population [39–41]. Substantial heterogeneity was observed among these studies, and none of them reported on all Pharyngeal Lumen Occlusive core metrics (PhCI, VCI, MCI, HCI) making it impossible to compare all our findings. However, one study published HRIM data of six laryngectomized patients without dysphagia swallowing 10 cc thin liquid boluses [41]. Compared to that paper, our laryngectomized participants exhibited lower VCI (96 vs. 272) and MCI (102 vs. 125) pressures. It is important to note that our results are measured in mmHg.cm.s, while the study we are comparing to, used a slightly different outcome measure (mmHg.s).

### Hypopharyngeal Intra-Bolus distension pressure

No significant changes were observed in the hypopharyngeal Intra-Bolus distension pressure (IBP). However, for thick liquid and solid boluses, the same trend as in the pharyngeal lumen occlusive pressure metrics was found in which the IBP decreased from T0 to T1 and then increased at T2. According to the literature, IBP corresponds to the pressure 1 cm superior of the UES apogee position at the time of maximum hypopharyngeal distension deduced from impedance [33]. In other words, impedance measurement relies on the bolus’ conductivity and enables the assessment of bolus flow and residue. In our cohort, the VFSS assessment combined with the %residue DIGEST scoring tool showed improvements in residue, with a decrease from 2 (‘*majority residue’*) at baseline (T0) to 1 (‘*less than half residue’*) at T1. This finding fits the finding of decreased IBP at T1 for thick liquid and solid.

In comparison with a study of healthy controls, our cohort showed higher IBP values than the controls (155 vs. 2.4mmHg). These extremely high IBP pressure in our participants might be due to their altered anatomy or could be caused by artefacts (e.g., contact of the HRIM catheter against the voice prosthesis). This raises the question of whether IBP is a feasible metric for assessing swallowing function in laryngectomized patients. Given the earlier mentioned studies [39–41], none of those reporting data on IBP, making it impossible to compare our results with those of other laryngectomized patients.

### UES relaxation and opening

The Relaxation Time (UES RT), known as the measure of the duration of the UES relaxation (reducing sphincter tone), and the Maximum Admittance (UES MaxAd), known as the core metric measuring the extent of UES opening, remained stable over time. The UES Integrated Relaxation Pressure (UES IRP) decreased across all bolus types between T0 and T1 but returned to baseline levels, or even exceeded them, at T2. Based on literature, higher UES IRP values indicate incomplete relaxation, which was not observed in our laryngectomized population. Instead, we saw a small improvement in relaxation at T1, which disappeared at T2. Compared to data on healthy individuals, the UES IRP in our laryngectomized group was lower (0.4 vs. 5.0 mmHg) [36]. This might be caused by the surgery since 13 of the 17 patients in this study had a myotomy of the upper esophageal sphincter muscle (see Table 1).

It would have been interesting to investigate whether there was a difference between laryngectomized patients with and without stenosis. However, only five participants diagnosed with stenosis were included in this study (S02, S03, S04, S09, and S14), and the T0 assessment for S14 was unavailable. As a result, there is insufficient data to analyze any potential differences in outcomes. At T1, participant S04 experienced severe stenosis, which may have contributed to the extremely high neopharyngeal pressures observed at that time point.

### Correlations

The correlation heat map in this study revealed moderate correlations between the two objective strength parameters, Jaw Opening and Chin Tuck. Additionally, we observed moderate correlations between the PhCI, IBP and UES IRP across the different consistency levels. Furthermore, there was a moderate correlation found between the PhCI solid and swallowing efficiency (VFSS, %residue DIGEST). However, no correlations were found between the different HRIM core metrics and the swallowing capacity (SPEAD) nor the muscle strengths (Chin Tuck and Jaw Opening). This was surprising because, at participant-level, ten of the 17 participants showed a decreased PhCI, increased swallowing capacity (SPEAD) and increased muscle strength on the Chin Tuck or Jaw Opening assessment [5].

On subjective parameters, only moderate correlations were found between two questionnaires (EAT10, SOAL) and the PhCI solid at T0 for solid food, which does not seem to be very relevant. No other correlations were detected. The moderate correlations between the various subjective outcome themselves have no bearing for this HRIM study.

HRIM has been previously used in head and neck cancer patients for various purposes, including evaluating therapies or interventions [14]. It has shown sensitivity in detecting changes in patients treated with pharyngeal tongue base augmentation. While VFSS measures showed no changes in aspiration, residue, or severity, HRIM revealed increased UES opening, with unchanged mesopharyngeal pressures [42].

In our cohort, the results were surprisingly contrary to expectations. While we observed improved objective swallowing efficiency, capacity and increased muscle strength during the Clinical Phase II trial, HRIM did not reveal significant occlusive intraluminal pressure changes. At T1, there was a slight decrease in pressure compared to T0, followed by an increase at T2, though the differences were small. This initial decrease between T0 and T1 may suggest that participants required less pressure and encountered less resistance during swallowing after their six-week training period. Another explanation for lower occlusive intraluminal pressures at T1 might be muscle fatigue after completing the training period.

However, anatomical changes following laryngectomy, such as reduced muscle mass in the (neo)pharynx, can make it more challenging to train the remaining muscles and measure swallowing pressures accurately. These changes might mean that participants compensate by relying more on higher-up structures or other compensatory muscles for strength and bolus propulsion.

Additionally, it is possible that the SEA2.0 exercises do not directly train the pharyngeal musculature, but instead focus on engaging other muscles and enhancing swallowing coordination, both of which are critical for swallowing efficiency. Another hypothesis is that frequent, resistance-based exercises may increase the elasticity of the pharyngeal wall and muscles, potentially influencing swallowing function.

Given these factors, drawing definitive conclusions from this study remains difficult, and all interpretations are speculative. Further research is needed to determine which of these hypotheses is correct.

### Limitations

This study has several limitations. First, our HRIM protocol slightly deviates from the recommendations in the literature [11]. We adapted the protocol by using only 10 cc bolus as a surrogate for the various boluses encountered in daily nutrition, also to minimize X-ray exposure. Furthermore, analyzing the HRIM data was sometimes challenging due to the visually different swallows resulting from the altered anatomy of the participants but also since no HRIM reference values are available for this patient population. The extremely high hypopharyngeal Intra-Bolus pressure (IBP) measured 1 cm above the uppermost (apogee) of the upper esophagus sphincter is now interpreted as an artifact of the voice prosthesis. We cannot confirm this, as all our patients had a voice prosthesis.

We did not observe significant changes in the HRIM metrics of this specific group of laryngectomized patients due to training. We have to keep in mind that this patient cohort was small, heterogeneous and at the extreme end of the alaryngeal dysphagia spectrum, making them possibly not representative for the entire total laryngectomy population. Therefore, it could still be valuable to use HRIM more frequently in clinical practice and research for diagnostic purposes (e.g. stenosis or hypertonicity) and to study swallowing therapy across this and other head and neck cancer patient populations and therapy types. For future research, it may also be valuable to explore the integration of artificial intelligence (AI) to streamline the analysis and improve reliability.

## Conclusion

This exploratory study analyzed HRIM data in laryngectomized patients at three time points to evaluate the effects of resistance-based swallowing rehabilitation. Training caused no significant changes in Pharyngeal Lumen Occlusive Pressure, hypopharyngeal Intra-Bolus Pressure, and UES Relaxation and Opening. As patients clinically and objectively did improve and muscles got stronger, we postulate that pharyngeal muscles related to the pressure are not the only muscles involved in swallowing after laryngectomy. Despite the altered anatomy HRIM provides valuable insights, and is suitable to further explore in this population as many questions remain unanswered.

## Supplementary Information

Below is the link to the electronic supplementary material.

Supplementary Material 1

## Data Availability

The dataset generated and analyzed during the current study are available from the corresponding author upon reasonable request.
