# Supplementary material for: Evaluation of post-laryngectomy dysphagia rehabilitation using High-Resolution impedance manometry: an exploratory study
Source: Eur Arch Otorhinolaryngol. 2025 Oct 3;282(11):5815–27. doi: 10.1007/s00405-025-09642-z (PMC12605583; doi:10.1007/s00405-025-09642-z)

**Appendix 1**

*The Pharyngeal Lumen Occlusive*

The Pharyngeal Lumen Occlusive pressure are generated by muscle contraction within the pharyngeal region and includes different metrics including the global Pharyngeal Contractile Integral (PhCI), Velopharyngeal Contractile Integral (VCI), Mesopharyngeal Contractile Integral (MCI) and Hypopharyngeal (HCI) Contractile Integral [11].

The PhCI is a global measure of pharyngeal contractile vigor within a space–time box on the pressure topography plot spanning from the Velopharynx superiorly to the upper margin of the UES. The PhCI is the mean pressure within this domain multiplied by duration (s) and length (cm) in units of mmHg.s.cm [11, 25]. Low scores suggest global weakness in the whole pharynx [11].

The VCI, MCI and HCI are measures of contractile vigor within a space-time box on the pressure topography plot spanning the Velo-, Meso or Hypopharyngeal region only. VCI, MCI and HCI are the mean pressure within one of these domains multiplied by duration (s) and length (cm) in units of mmHg.s.cm [11, 26]. Low scores suggest weakness in the Velopharynx, Mesopharynx or Hypopharynx [11].

*The Hypopharyngeal Intra-Bolus Distension Pressure*

Hypopharyngeal Intra-Bolus pressure (IBP) is a marker of UES restriction to bolus flow during swallowing. The hypopharyngeal IBP is defined by the pressure 1 cm superior of UES apogee position at the time of maximum hypopharyngeal distension deduced from impedance topography in units of mmHg [11, 27-29]. High IBP suggests an increased pressure gradient across the pharyngo-esophageal junction and reduced UES opening, which may lead to compensatory changes in upstream functions, such as increased Mesopharyngeal or Velopharyngeal pressure generation [11].

The *UES Relaxation and Opening* metrics

UES relaxation refers to the neuromuscular process of reducing sphincter tone, while UES opening is the physical widening of the sphincter, which depends on both relaxation and hyolaryngeal movement for effective bolus passage. Therefore, the measurement of the UES is divided into relaxation and opening metrics. For relaxation, metrics include the UES Integrated Relaxation pressure (UES IRP) and UES Relaxation Time (UES RT). The UES IRP is a measure of the extent of UES relaxation. UES IRP is the median of the lowest non-consecutive 0.20–0.25 s of e-sleeve pressure in units of mmHg [11, 26, 28]. Higher UES IRP suggest incomplete UES relaxation [11]. The UES Relaxation Time (UES RT) is a measure of the duration of UES relaxation. UES RT is the e-sleeve pressure interval below 50% of baseline or 35 mmHg, whichever is lower, in units of sec [11, 30, 31]. Shorter UES RT seconds suggest shorter opening period of the UES [11].

UES opening can be measured by the metric UES Maximum Admittance (UES MaxAd). The UES MaxAd is a measure of extent of UES opening; with Admittance being the inverse product of Impedance. UES MaxAd is the highest admittance value recorded during trans-sphincteric bolus flow in units of millisiemens (mS) [11, 32-34]. Lower mS suggest reduced UES opening [11].

**Appendix 2**


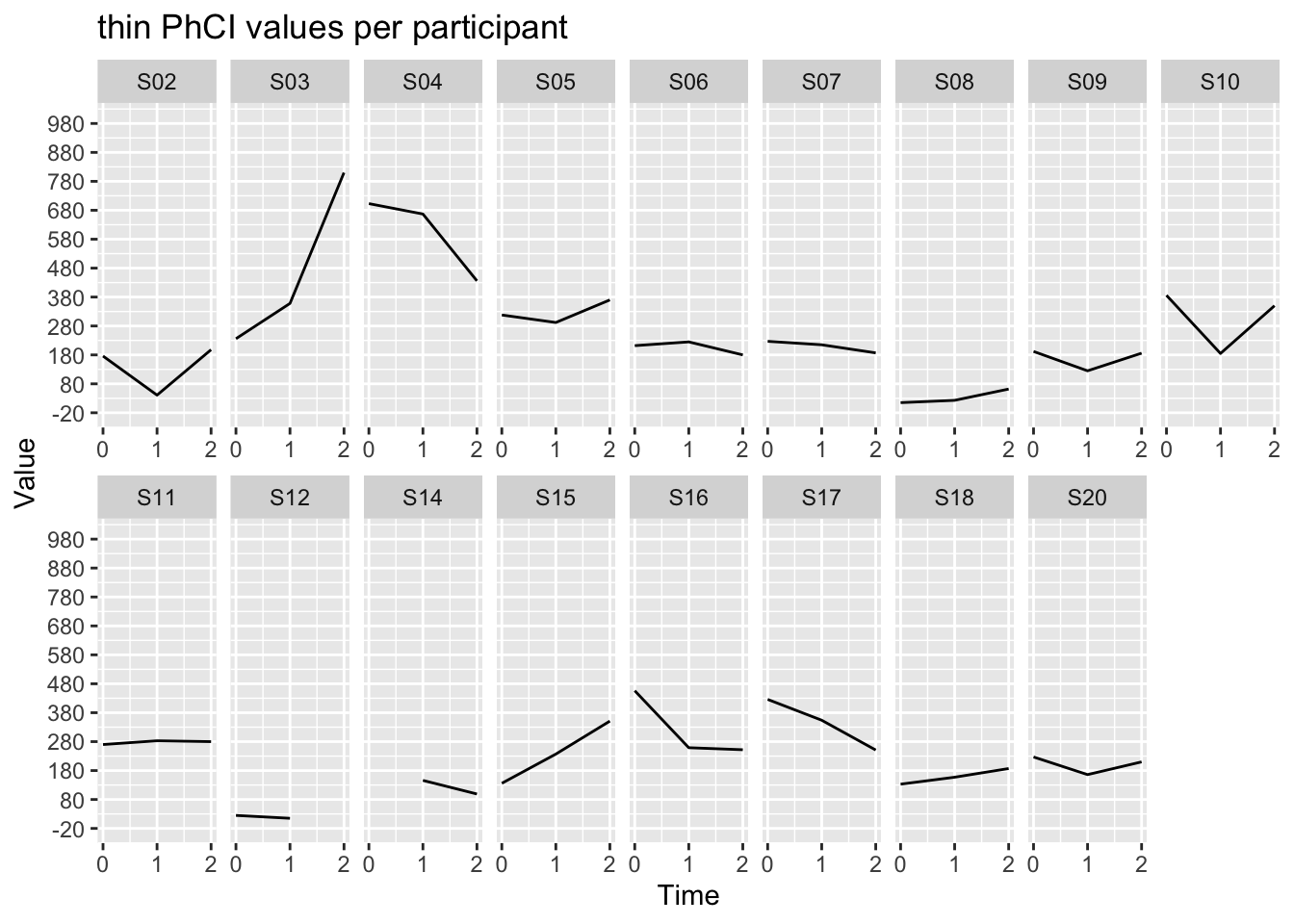

Supplement: Supplementary file 1 — Supplementary Material 1 [file 405_2025_9642_MOESM1_ESM.docx]
